# Off-season circulation and characterization of enterovirus D68 with respiratory and neurological presentation using whole-genome sequencing

**DOI:** 10.3389/fmicb.2022.1088770

**Published:** 2023-02-09

**Authors:** Hayley Cassidy, Erley Lizarazo-Forero, Leonard Schuele, Coretta Van Leer-Buter, Hubert G. M. Niesters

**Affiliations:** The University of Groningen, University Medical Centre Groningen, Department of Medical Microbiology and Infection Prevention, Division of Clinical Virology, Groningen, Netherlands

**Keywords:** enterovirus D68, respiratory infection, neurological infection, whole-genome sequencing, long-read sequencing

## Abstract

To explore an off-season enterovirus D68 (EV-D68) upsurge in the winter season of 2019/2020, we adapted a whole-genome sequencing approach for Nanopore Sequencing for 20 hospitalized patients with accompanying respiratory or neurological presentation. Applying phylodynamic and evolutionary analysis on Nextstrain and Datamonkey respectively, we report a highly diverse virus with an evolutionary rate of 3.05 × 10^−3^ substitutions per year (entire EV-D68 genome) and a positive episodic/diversifying selection with persistent yet undetected circulation likely driving evolution. While the predominant B3 subclade was identified in 19 patients, one A2 subclade was identified in an infant presenting with meningitis. Exploring single nucleotide variations using CLC Genomics Server showed high levels of non-synonymous mutations, particularly in the surface proteins, possibly highlighting growing problems with routine Sanger sequencing for typing enteroviruses. Surveillance and molecular approaches to enhance current knowledge of infectious pathogens capable of pandemic potential are paramount to early warning in health care facilities.

## 1. Introduction

Enterovirus D68 (EV-D68) has been increasingly recognized as an emerging virus, with outbreaks occurring primarily in children (Piralla et al., 2018; Kamau et al., 2019; Andrés et al., 2022). First reported in 1962 in California, EV-D68 was sporadically reported worldwide until 2013, with small outbreaks described in the Philippines (2009–2011; Imamura and Oshitani, 2015), Japan (2010; Hasegawa et al., 2011), United States of America (USA; 1970–2005; Khetsuriani et al., 2006) and in the Netherlands (2010; Rahamat-Langendoen et al., 2011). The largest known outbreak to date was reported in the USA in 2014, with over a thousand cases in children presenting with severe respiratory disease (Midgley et al., 2015). During the 2014 outbreak, a number of EV-D68 cases were linked to acute flaccid myelitis (AFM), presenting with asymmetric flaccid limb weakness and cranial nerve dysfunction (Messacar et al., 2015; Vogt and Crowe, 2018). In the same year, EV-D68 was also reported in Europe, Southeast Asia and Chile (Holm-Hansen et al., 2016). Since 2014, EV-D68 outbreaks have occurred in a biennial pattern between 2016 and 2018, typically in even years in temperate climates (Howson-Wells et al., 2022) and emerging most frequently in the USA, Europe, Argentina and Taiwan (Knoester et al., 2017; Hu and Chang, 2020). While EV-D68 typically peaks in late summer and autumn in temperate climates (Hodcroft et al., 2022), an off-season upsurge was observed across Europe during the 2019–2020 winter season (Midgley et al., 2020).

EV-D68 has been shown to have substantial genetic diversity (Kramer et al., 2018; Howson-Wells et al., 2022). Sequences can be separated into three main clades: A, B, and C, which can be further divided into A1–A2 and B1–B3 subclades (Hodcroft et al., 2022). The EV-D68 genome is approximately 7,500 nucleotides in length and encodes a single polyprotein which consists of four structural proteins (viral proteins 1–4) and seven non-structural proteins (2A–C and 3A–D; Eshaghi et al., 2017; Dyrdak et al., 2019). Generally, EV-D68 sequences have been investigated using the hypervariable viral protein 1 (VP1) gene, which is present on the viral capsid and has been used as a target for subtype differentiation (Harvala et al., 2018). Since 2014, subclades B1 and B3 have become dominant (Hodcroft et al., 2022). The emergence of the A2 subclade was additionally reported in East Asia in 2016 (Midgley et al., 2020). By 2018, clusters of subclades B3 and A2 appear to be predominating worldwide.

Whole-genome sequencing (WGS) has been used previously to investigate the diversity and evolution of EV-D68 during the 2014 and 2016 outbreaks (Dyrdak et al., 2019). Different subclades have been identified in respiratory and neurological samples, with varying rates of amino acid substitutions observed (Zhang et al., 2016; Wang et al., 2017; Hodcroft et al., 2022). In this study, we obtained near-complete EV-D68 sequences from patients presenting with respiratory or neurological disease at a regional university hospital in the Netherlands during the winter season of 2019–2020. Using WGS, we aimed to explore our patient cohort, along with the circulating clades responsible for the rise in cases with the intention of contributing to a greater understanding of potential evolutionarily changes within EV-D68.

## 2. Materials and methods

### 2.1. Patient selection

Patients were selected following a positive EV-D68 detection through our laboratory developed test (LDT) real-time reverse transcriptase PCR (RT-qPCR; Poelman et al., 2015). Sanger sequencing targeting the VP1 gene (approx. 326 bp; Nix et al., 2006) was performed on samples with a Ct value below 32. Sanger sequencing data was analyzed in BioNumerics v6.1, while patient information was extracted from the electronic patient database system.

### 2.2. Nucleic acid extraction

All samples were centrifuged at 6,000xg for 2 min. A total of 190 μL of supernatant was used as input for extraction on the easyMAG (bioMérieux, Inc., Marcy l’Etoile, France), which was eluted in 110 μL. Lysis buffer served as a negative control. A total of 70 μL of isolated nucleic acids were cleaned and concentrated to 40 μL using the RNA clean and concentrator kit-5 (Zymo Research, Irvine, USA), including an in-column DNase treatment using TurboDNase (Thermo Fisher Scientific, Waltham, USA), according to the manufacturer’s recommendations.

### 2.3. cDNA synthesis

Near full-length amplification was achieved using a one-step RT PCR and four overlapping fragments (approximately 2,000 bp) from primers designed by Dyrdak and colleagues (Dyrdak et al., 2019). Briefly, four separate reactions for each overlapping fragment contained; 1 μL of Superscript III RT/Platinum Taq HiFi Enzyme mix (Invitrogen, Stockholm, Sweden), 25 μL of 2× reaction mix, 2.5 μL of forward (0.5 μM) and reverse (0.5 μM) primers, 0.5 μL of random hexamers (1 ng/μL; Thermo Fisher Scientific) and 8.5 μL of RNase-free water. Finally, 10 μL of RNA template was added per fragment for each sample to attain a total reaction volume of 50 μL. We adjusted the PCR cycling conditions to account for the increased cDNA input required by Oxford Nanopore Technologies (ONT) ligation library preparation kit: 30 min at 50°C, 2 min at 94°C, ×33 (15 s at 94°C, 30s at 50°C, 2 min at 68°C), 5 min at 68°C, ∞ at 4°C. Each fragment was adjusted to 25 ng and pooled to achieve a total of 100 ng for each sample. cDNA fragments with <25 ng were re-amplified using the PCR reaction stated above with a modified PCR cycling condition to account for cDNA as an input: 1 min at 98°C, ×29 (10s at 98°C, 30s at 50°C, 3 min at 72°C), 5 min at 72°C, ∞ at 4°C.

### 2.4. Library preparation and sequencing

Sequencing libraries were generated using the Ligation Sequencing Kit (SQK-LSK109; ONT, Oxford, United Kingdom) and native barcoding expansion (EXP-NBD104; ONT). The One-pot protocol was followed for native barcoding for amplicons (Josh Quick, 2020)^1^. The libraries were pooled by equal mass and 25fM was loaded on FLO-MIN106 R9.4.1 flow cell on a MinION device for long-read sequencing (ONT).

### 2.5. Assay validation

An EV-D68 culture from the National Institute for Public Health and the Environment (RIVM) was used to validate the workflow prior to running the clinical samples (Supplementary materials and methods 1.1 and Supplementary Figure S1).

### 2.6. Data analysis

Sequencing reads were first base-called with Guppy v6.0.1 (ONT) with high accuracy mode enabling the “trim barcodes,” “barcode both ends” and “mid-read barcode filtering” option (Supplementary Table S1). Subsequent reads were uploaded onto the CLC Genomics Server 21.0.5 (CLC; Qiagen, Aarhus, Denmark). Only reads with >300 bp were kept and mapped against the human genome (hg19) to filter out human reads. To create a reference database, a total of 893 near-full length EV-D68 reference sequences (7,000–8,000 bp) were downloaded from the Virus Pathogen Resource (Supplementary materials and methods 1.2). The trimmed reads were mapped against this reference database using an 70% nucleotide identity and 80% length fraction on CLC to determine a best hit reference for each sample (Supplementary Table S2). Consensus sequences were extracted with a coverage cut-off of >30x (Karst et al., 2021), with confirmation using NCBI BLAST and uploaded onto the online enterovirus typing tool (v0.1) to obtain sub genogroups.^2^

### 2.7. Phylogenetic and evolutionary analysis

Patient consensus sequences and 893 reference genomes were aligned with MAFFT (v7.471; see Supplementary materials and methods 1.2). A time-scaled phylogenetic analysis using the augur pipeline (v7.0.2) and TimeTree v0.8.1 (Sagulenko et al., 2018) implemented in Nextstrain (Hadfield et al., 2018) was performed (Supplementary Table S3). A maximum likelihood tree was inferred using a GTR + gamma distribution, a strict molecular clock and a coalescent Skyline tree prior (Supplementary Table S3). The tree was rooted using GenBank accession number AY426531 (1962 Fermon strain). The time-scaled tree was then visualized using auspice (v0.8.0; Hadfield et al., 2018) and detailed with country of origin. To explore the temporal signal and heterochronous data, a root-to-tip regression analysis on TempEst (v2.7.0; Rambaut et al., 2016) was applied for the maximum likelihood tree without a molecular clock. To determine the type of selection pressure, a Mixed Effects Model of Evolution (MEME; Murrell et al., 2012) was applied on Datamonkey (v1.6.0) using two MAFFT alignments, one including the 824 EV-D68 references (see Supplementary materials and methods 1.2) and patient sequences, and the other with only patients sequences (CDS regions only; posterior probability [PP] value = 0.05; Supplementary Table S4). Finally, single nucleotide variation (SNV) was investigated within the patient sequences using a Fixed Ploidy algorithm with 90% variant probability and 90% minimum frequency on CLC, with 100x minimum coverage and a Q score of 30 (Supplementary Table S5).

### 2.8. Ethics statement

Oral consent for the use of clinical samples for research purposes is routinely obtained upon patient admission at the University Medical Center Groningen (UMCG), in accordance with the guidelines of the Medical Ethics Committee. All experiments were performed in accordance with the guidelines of the Declaration of Helsinki and all samples were anonymized. A waiver was obtained by the UMCG Ethics Committee: METc 2009.169. The sequencing data has been deposited in the Sequence Read Archive under the BioProject number: PRJNA865246.

## 3. Results

### 3.1. Off-season upsurge

**Figure 1:**
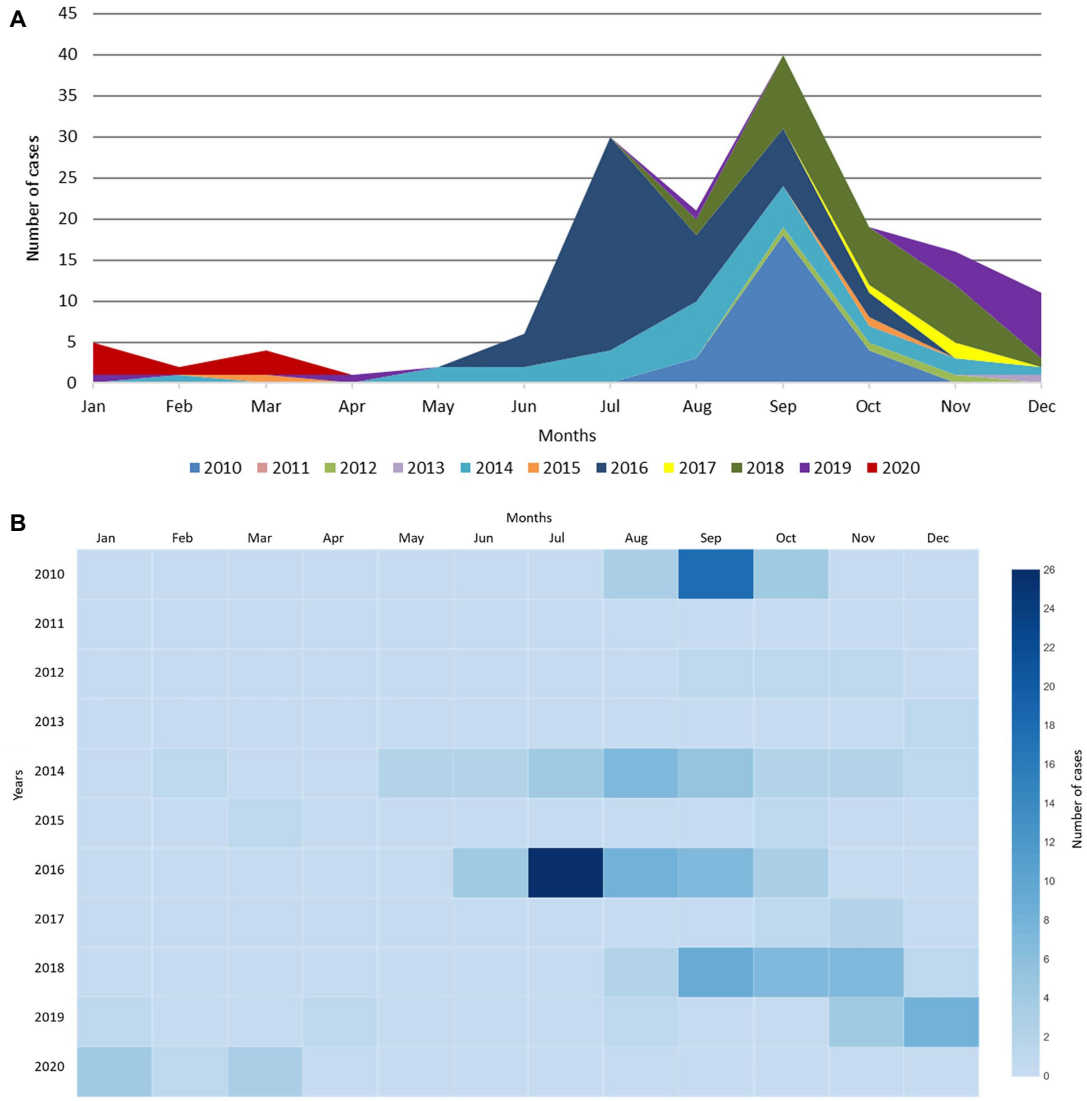
EV-D68 detection from January 2010 to March 2020 at the UMCG. **(A)** EV-D68 detection per month at the UMCG between 2010 and 2020. A distinct rise can be observed which does not follow the usual trend, shown in purple (2019) and red (2020). **(B)** Heatmap of EV-D68 detection per month at the UMCG between 2010 and 2020.

From January 2010 to March 2020, a total of 157 EV-D68 detections were recorded at the UMCG, typically peaking between July and September (Figure 1), with the highest number of cases in July 2016 (*n* = 26 cases). Since 2014, there appears to be a general shift in the number of reported cases toward the late autumn months (Figure 1B). By the winter season of 2019–2020, a distinct rise in the number of cases (*n* = 20) can be observed, which is both off-season and in an odd year, with a peak in December 2019 (*n* = 8 detections; Figures 1A,B).

### 3.2. Off-season EV-D68 clinical characterization

The clinical characteristics of the 20 patients with an EV-D68 detection during the 2019/2020 off-season upsurge are shown in Table 1. For four patients (1–3 and 16), only birth date, gender and sample collection information were recorded. Sample 16 had additional diagnosis information.

**Table 1:** Clinical characteristics of patients with an enterovirus D68 detection.

Patient sample number	Sex	Age (years)	Co-morbidities	Clinical presentation	LOS (days)	Diagnosis	Co-infections	Patient management
EVD68_GR_01_22.11.19	M	67	NA	NA	NA	NA	NA	NA
EVD68_GR_02_23.11.19	F	5	NA	NA	NA	NA	NA	NA
EVD68_GR_03_23.11.19	F	50	NA	NA	NA	NA	NA	NA
EVD68_GR_04_28.11.19	F	22	Cystic fibrosis, diabetes	Respiratory	8	URTI	*P. aeruginosa*	Tobramycin and colistin
EVD68_GR_05_02.12.19	M	64	Acute myeloid leukemia, allogenic SCT	Respiratory	10	Pneumonia	HSV-1, RV, aspergillosis and *H. influenzae*	Ceftriaxone, amoxicillin, voriconazole and valaciclovir
EVD68_GR_06_08.12.19	M	69	Severe aortic valve stenosis	Respiratory	13	Not specified	RV	None
EVD68_GR_07_09.12.19	F	5	None	Respiratory	2	Pneumonia	None	NSAID
EVD68_GR_08_11.12.19	F	0	Atelectasis, pre-mature	Respiratory	48	Bronchiolitis^a^	*H. influenzae*	Amoxicillin and azthiromycin. Oxygen *via* Optiflow
EVD68_GR_09_14.12.19	M	0	Down syndrome	Respiratory	5	EV Bronchiolitis	*Moraxella catarrhali*s and *H. influenzae*	Oxygen *via* nasal prongs, ceftriaxone
EVD68_GR_10_14.12.19	F	5	Adenoid hypertrophy, ex-premature	Neurological	9	EV-AFM	None	IVIG, cefotaxime and amoxicillin + clavulanic acid
EVD68_GR_11_17.12.19	F	2	Ex-premature	Respiratory	2	URTI	RSV (Ct15), RV (Ct29), CoV (Ct35), HPIV-1 (Ct34), HPIV-4 (Ct36) and AV (Ct30)	Augmentin, oxygen *via* Optiflow
EVD68_GR_12_25.12.19	M	0	Anaemia	Fever	3	EV Meningitis	None	Hydrocortisone
EVD68_GR_13_13.01.20	M	16	Premature, Psychomotor retardation, epilepsy, PVL	Respiratory	5	Bronchitis	None	Oxygen *via* Optiflow
EVD68_GR_14_20.01.20	M	5	None	Respiratory	10	URTI and bronchial obstruction	RSV type B	Salbutamol, oxygen *via* Optiflow, prednisone and amoxicillin + clavulanic acid
EVD68_GR_15_21.01.20	F	1	KHE with KMP	Respiratory	3	URTI	None	None
EVD68_GR_16_29.01.20	F	1	NA	NA	NA	EV AFM	NA	NA
EVD68_GR_17_19.02.20	M	67	Epilepsy, type 1 diabetes mellitus, lung TX	Respiratory, atypical cardiac complaints	20 (till death)	Pneumonia^b^	*P. aeruginosa*	Piperacillin/ tazobactam and prednisolone
EVD68_GR_18_12.03.20	M	0	Premature	Fever	2	Neonatal cholestasis	None	None
EVD68_GR_19_13.03.20	F	0	Ventricular septal defect, prematurity, PDA, PVL Gd 1, neonatal IVH	Respiratory and diarrhea	40 (till death)	Severe Broncho-Pulmonary Dysplasia	None	Dexamethasone, palliative care
EVD68_GR_20_19.03.20	F	0	Prematurity, PVL Gd 1	Fever and Respiratory	2	URTI	None	None

a Enterovirus respiratory tract infection with bacterial superinfection.

b Infection from *Pseudomonas aeruginosa* infection. Antibiotics were only given to adults who were subsequently diagnosed with either a primary or secondary bacterial infection.

LOS, length of stay; NA, not available; EV, enterovirus; AFM, acute flaccid myelitis; IVIG, Intravenous Immunoglobulin; SCT, Allogeneic Stem Cell Transplantation; TX, transplant; HSV, herpes simplex virus; NSAID, Non-steroidal Anti-inflammatory Drugs; RV, rhinovirus; RSV, Respiratory syncytial virus; CoV, coronavirus; HPIV, Human parainfluenza virus; AV, adenovirus; HSV, Herpes simplex virus; PVL, Periventricular leukomalacia; IVH, Intraventricular hemorrhage; URTI, Upper respiratory tract infection; KHE, Kaposiform hemangioendothelioma; PDA, Patent ductus arteriosus; KMP, Kasabach–Merritt phenomenon; Gd, Grade; IVH, Intraventricular hemorrhage; *M. catarrhalis*, *Moraxella catarrhalis*; *H. influenzae*, *Haemophilus influenzae*; *P. aeruginosa*, *Pseudomonas aeruginosa*.

A total of 13 children (<16 years of age; median 1 year) and seven adults (median 64 years) were included in the study. Eight children (61%) were found to have an underlying medical condition, of which prematurity had the highest occurrence (*n* = 6; Table 1). All adults with available clinical information (*n* = 5) had at least one underlying condition. Females accounted for 55% of the patient population (*n* = 11).

Of the eight children with a respiratory infection, four had a short length of stay (LOS; 2–3 days), one had a medium LOS (4–7 days) and three had a long LOS (>7 days). According to the attending clinician, EV-D68 was found to be the causative agent in five of the eight children. Of the four adults with a respiratory infection, one had a medium LOS and three had a long LOS (one adult died after 20 days in hospital). According to the attending clinician, EV-D68 was found to be the causative agent in only one adult (Table 1). Of the three children with an EV-D68 neurological infection, one child (<1 year) was diagnosed with meningitis and two children (5 years and 1 year) were diagnosed with AFM. The 5-year-old child was given antibiotics and intravenous immune globulin (IVIG) treatment prior to their AFM diagnosis. Within this small off-season cohort, there was no EV-D68 neurological presentation reported in adults.

### 3.3. EV-D68 whole-genome sequencing

Seventeen respiratory, two cerebrospinal fluid (CSF), and one fecal sample were included for WGS. Four samples (20%) had an unsuccessful Sanger sequencing typing result previously. Twenty near-full genomes were recovered (genome size range 7,173–7,222 bp), indicating a robust approach (Table 2). All but one sample (patient number 5) had sufficient sequencing depth (59.4×–66,459.4×) for SNV calling and phylogenetic analysis. Supplementary Figure S2 illustrates an example of the genome coverage pattern achieved.

**Table 2:** Metatable of sample information.

Patient sample number	Sample type	EV RT-qPCR Ct	Date of collection	Sanger sequencing result	Best reference on NCBI	Best reference on NCBI (size bp)	Genome coverage (%)	Average sequence depth (x)
EVD68_GR_01_22.11.19	Respiratory	24	22/11/2019	EV-D68	MN245405	7,177	99.92	53,618.23
EVD68_GR_02_23.11.19	Respiratory	20	23/11/2019	EV-D68	MN726800	7,331	98.24	9,435.15
EVD68_GR_03_23.11.19	Respiratory	20	23/11/2019	EV-D68	MN246019	7,324	98.36	411.58
EVD68_GR_04_28.11.19	Respiratory	23	28/11/2019	EV-D68	MN246019	7,324	98.58	32,104.29
EVD68_GR_05_02.12.19	Respiratory	29	02/12/2019	EV-D68	MN245983	7,324	98.24	59.41
EVD68_GR_06_08.12.19	Respiratory	17	08/12/2019	EV-D68	MN246018	7,323	98.35	19,748.44
EVD68_GR_07_09.12.19	Respiratory	24	09/12/2019	EV-D68	MN245983	7,324	98.40	28,357.59
EVD68_GR_08_11.12.19	Respiratory	18	11/12/2019	EV-D68	MK419050	7,281	98.86	13,029.08
EVD68_GR_09_14.12.19	Respiratory	19	14/12/2019	EV-D68	MN245983	7,324	98.40	25,355.01
EVD68_GR_10_14.12.19	CSF	31	14/12/2019	Untypeable	MN365200	7,331	98.25	18,952.83
EVD68_GR_11_17.12.19	Respiratory	26	17/12/2019	Untypeable	MN246039	7,324	98.40	20,796.99
EVD68_GR_12_25.12.19	CSF	27	25/12/2019	EV-D68	MG757146	7,297	99.00	25,950.36
EVD68_GR_13_13.01.20	Respiratory	19	13/01/2020	EV-D68	MN246019	7,324	98.48	47,391.30
EVD68_GR_14_20.01.20	Respiratory	19	20/01/2020	EV-D68	MN245983	7,324	98.43	59,811.59
EVD68_GR_15_21.01.20	Respiratory	26	21/01/2020	Untypeable	MK105981	7,326	97.90	47,521.93
EVD68_GR_16_29.01.20	Respiratory	31	29/01/2020	Untypeable	MN245983	7,324	98.02	21,979.54
EVD68_GR_17_19.02.20	Respiratory	32	19/02/2020	Not typed	MN245983	7,324	98.14	131.83
EVD68_GR_18_12.03.20	Respiratory	15	12/03/2020	EV-D68	MN245983	7,324	98.31	11,206.88
EVD68_GR_19_13.03.20	Fecal	19	13/03/2020	EV-D68	MN245983	7,324	98.42	66,459.41
EVD68_GR_20_19.03.20	Respiratory	11	19/03/2020	EV-D68	MK105981	7,326	98.35	29,310.58

CSF, cerebrospinal fluid; EV-D68, enterovirus D68; RT-qPCR, reverse transcriptase real-time PCR; Ct, cycle threshold. The average read length for each patient sample is presented in Supplementary Table S6. Accession numbers for the patient consensus sequences can be found under the BioProject number: PRJNA865246.

### 3.4. Phylogenetic analysis

**Figure 2:**
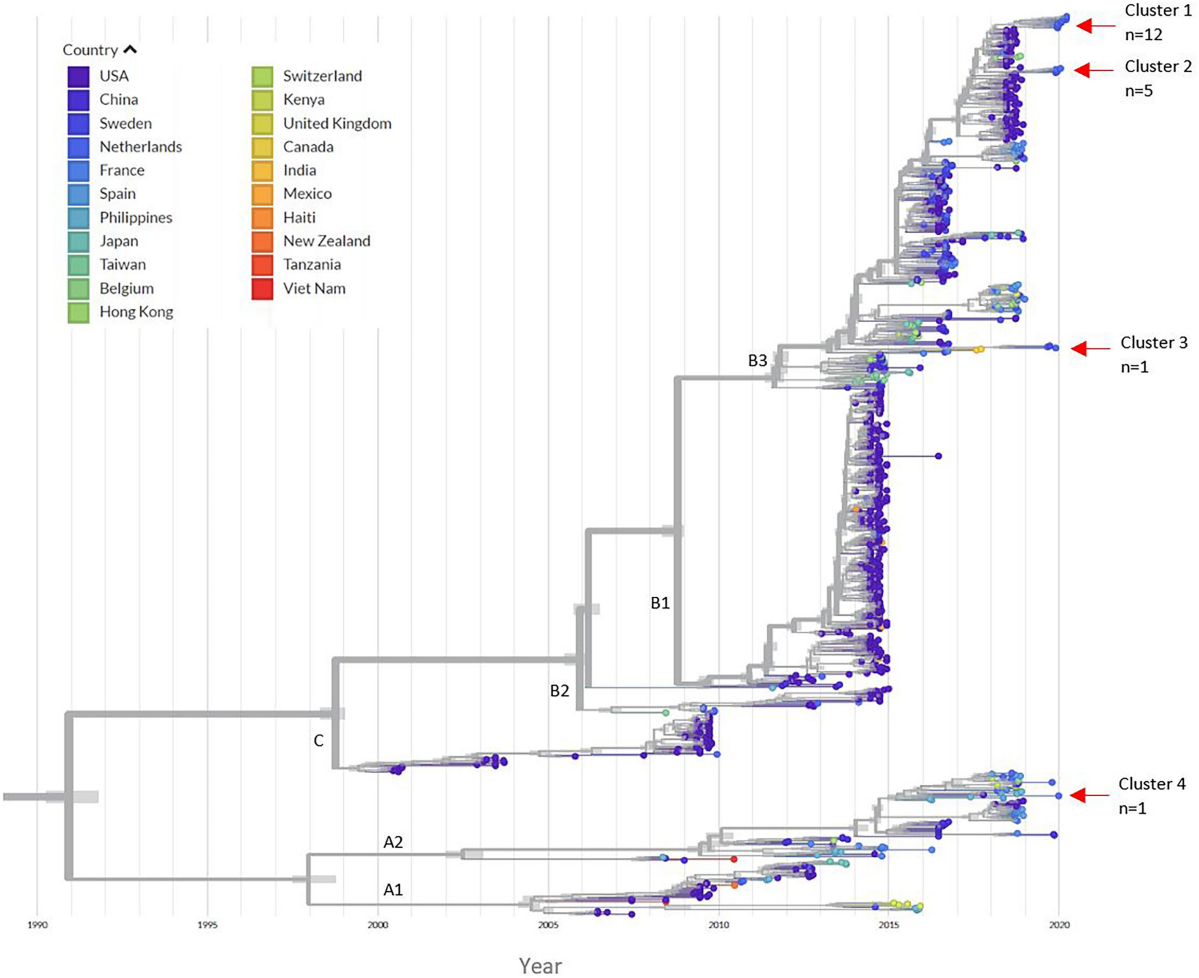
Time-scaled phylogenetic tree. The left panel indicates the corresponding countries to each EV-D68 sequence in the tree. The red arrows indicate the location of the four different clusters of our 2019/2020 patient samples. From the 892 references downloaded; 21 sequences were manually removed due to poor quality after the initial alignment (Supplementary Table S8). A total of 872 sequences were run through the Nextstrain pipeline. An additional 29 references were removed after a second alignment. The Nextstrain pipeline automatically removed problematic sequences during tree refinement (*n* = 12 references) and uploading to auspice (*n* = 7 references), this included EVD68_GR_05_02.12.19 (Supplementary Table S8). A total of 824 EV-D68 references were included in the phylogenetic analysis. Supplementary Figures S4, S5 illustrate zoomed in images of the clusters. The time-scaled tree had an estimated evolution rate of 3.05 × 10^−3^.

Phylodynamic analysis indicated that 18 sequences from the off-season upsurge clustered within three distinct subgroups within the B3 lineage, with a most recent common ancestor (MRCA) estimated to have occurred in late 2018, following a period of high genetic diversity in mid-2017 (Figure 2; Supplementary Figures S4, S5). Clusters 1 and 2 had the highest similarity to strains from the USA collected in 2018, with 99% (98.9%–99.0%) and 98.9% (98.85%–99.1%) identities, respectively. Cluster 3 had highest similarity to strains from the Netherlands (99.4%) and Sweden (98.87%), collected in 2019. Patient 12 (Cluster 4) clustered within the A2 lineage with a USA strain collected in 2017 (97.90% identity). To initially estimate the evolutionary changes of EV-D68, the temporal signal was explored using a root-to-tip regression analysis (Supplementary Figure S3; Supplementary Table S7). A strong association was observed between genetic distances and collection dates, highlighting heterogeneity between the EV-D68 sequences (*R*^2^ = 0.9544). Applying a time-scaled tree using the Nextstrain pipeline, we estimated the evolutionary rate for the entire EV-D68 genome to have 3.05 × 10^−3^ substitutions per site per year.

### 3.5. Evolution analysis

**Figure 3:**
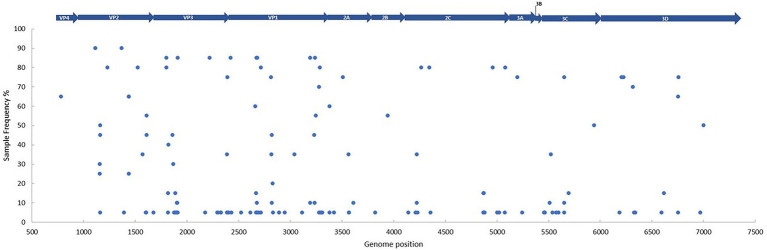
The frequency and distribution of single nucleotide variants per genome position between the patient samples (*n* = 20) and the Fermon strain (accession number AY426531). Every blue dot represents a non-synonymous change on the sequence, with the gene position depicted above. Sample frequency denotes the percentage of samples in the data set with the non-synonymous change. A Fixed Ploidy algorithm was applied on CLC Genomics Server v21.0.5 using the following parameters: 90% variant probability, 90% minimum frequency, min 100x coverage, max 100,000x coverage, Q-score threshold = 30.

SNV’s (non-synonymous mutations only) were explored within all 20 patient samples, with the 1962 Fermon stain (accession number AY426531) used as a reference to allow comparisons with other studies (Dyrdak et al., 2019). Non-synonymous mutations were scattered across the genome, both in structural and non-structural segments of the polyprotein, except in the 3B gene (essential for replication and comprises of 0.88% of genome). We observed a total of 836 non-synonymous mutations (in relation to the Fermon strain), with VP1 containing the highest number of variants (*n* = 257; Figure 3; Supplementary Table S9). All variants detected were unique per genome position, and therefore we can report one EV-D68 infection per patient.

Overall, 44 multiple and 92 single nucleotide variants were observed, with particularly high activity within the BC loop region (position 2,659–2,698) in the VP1 gene. The following frame-shift mutations in the VP1 gene were of note: one deletion (removal of GA [His651] in position 2,679–2,680), two replacements (replacement of GGG to A [Gly649] in position 2,677–2,679 and C to TGA [Ser647] in position 2,670) and one insertion (TA [ser647] in position 2,669–2,670). All non-synonymous mutations for each sample, along with their corresponding regions are detailed in Supplementary Table S9. Although no shared variants were observed amongst all the off-season sequences, 18 did share variants in two regions (with 90% frequency) present in the VP2 gene (Figure 3; Supplementary Table S9). No obvious variant patterns were observed in patients with CSF collection (Supplementary Table S9) or neurological presentation (Supplementary Figure S6).

**Figure 4:**
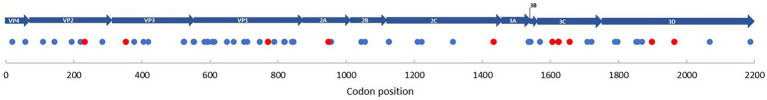
Selection pressure on individual codon sites. Blue dots represent the selection pressure on individual codon sites for all reference genomes (*n* = 824), along with EV-D68 patients (*n* = 19; 1962–2020). Red dots represent the selection pressure on individual codon sites for only EV-D68 patients (*n* = 19). MEME was used to determine diversifying selection (PP value < 0.05). Individual sites are specifically indicated in Supplementary Figure S10.

To determine any potential changes in the type of selection pressure during our off-season upsurge, two MEME models were used to explore the distribution of variation at the codon level within the EV-D68 population (*n* = 824) and the patient sequences (*n* = 19; Figure 4). Exploring the whole population (shown in blue), we found evidence of episodic or diversifying selection at 54 codon sites (PP value = 0.05 [occurring 95%]), scattered throughout the genome, with high clustering within the viral capsid genes. Exploring within the patient population (shown in red), we found evidence of episodic or diversifying selection at 10 codon sites, with the highest clustering in the 3C gene, followed by the capsid genes.

## 4. Discussion

In this study, we provide clinical and phylogenetic information from hospitalized patients from an off-season EV-D68 upsurge in the winter season of 2019–2020. Using a modified WGS approach, we were able to obtain near-complete EV-D68 genomes from a wide range of Ct values, including Ct values >30 and sample material, including CSF. The summer/autumn seasonal circulation is well recognized for all enteroviruses in temperate climates, with a biennial recurrence of EV-D68 observed in North America and Europe (Elrick et al., 2021; Fall et al., 2022). Interestingly, our data shows a general shift towards the later months, with the upsurge emerging in an odd year (Figure 1). This off-season occurrence was also observed by other European countries (Midgley et al., 2020). Although the seasonality of enteroviruses is likely determined by environmental factors such as temperature and air humidity (Fares, 2013; Pons-Salort et al., 2018), the biennial occurrence of EV-D68 is thus far not understood. While factors including herd immunity, human behavior and circulation of other (entero-) viruses may play a part, the role and occurrence of viral variants requires more investigation.

The majority of patients in our study had underlying conditions, with prematurity highest among children and typically multiple comorbidities in adults, echoing previous studies (Lau et al., 2016; Fall et al., 2022). Overall, we observed 13 cases (65%) of respiratory illness and three cases (15%) of neurological illness (all in children; Table 1), two of which had AFM and one had EV-D68 meningitis. In our small cohort, we found a relatively high number of cases requiring admission to hospital, with 75% of patients admitted for over 2 days. This most likely reflects the tertiary-care character of our hospital. A similar ratio of severe illness was additionally observed within Europe during the same time period (Midgley et al., 2020). Interestingly, we identified one A2 subclade from a child (<1 year) with meningitis. Although detection of EV-D68 in CSF is rare (Fares, 2013), to the best of our knowledge, we report the first detection of an A2 subclade associated meningitis in CSF. EV-D68 was also detected from one fecal sample from a patient presenting with respiratory and diarrhea illness. Although EV-D68 detection from fecal samples is uncommon, it could provide an alternative for widespread population surveillance studies (Corpuz et al., 2020; Tedcastle et al., 2022). It is important to note that the true burden and circulation within the community is difficult to estimate, given that community surveillance is not performed and subtype differentiation is typically only performed for severer cases following clinical consultation.

Genotyping enteroviruses is important for understanding changes in epidemiology, which could aid outbreak preparedness and patient management (Harvala et al., 2018). WGS has been previously used to explore viral dynamics, particularly during outbreak scenarios and especially during the SARS-CoV-2 pandemic (Oude Munnink et al., 2021). We adapted a previously applied EV-D68 WGS method for ONT platforms to characterize our upsurge samples, including previously untypeable samples.

Using the Nextstrain pipeline, we could perform more real-time tracking of EV-D68 evolution and spread overtime (Figure 1). A high genetic diversity can be observed among the available EV-D68 sequences, indicating diversification and persistence even in low circulation years, similarly reported by Hodcroft et al. (2022). Interestingly, there appears to be continuous emergence in conjuncture with clade replacement prior to 2018, with the rise of the predominating B3 subclade. In our study, 18 samples clustered with the B3 subclade within three different branches, suggesting introduction at several different timepoints in the Netherlands, followed by local spread. Subclade B3 exhibits high levels of geographical circulation (and mixing) since its emergence in 2016 (Wang et al., 2017), having been found in several countries globally (Midgley et al., 2020). The detection of the A2 subclade additionally suggests an ongoing low-level (and potentially undetected) transmission in Europe. Given their increasing observation between 2014 and 2020 (Lau et al., 2016; Midgley et al., 2020; Hodcroft et al., 2022), it could render this finding potentially important for monitoring. Moreover, although the majority of sequences from our study appear to cluster with USA strains, as some countries deposit more sequences than others, it could also result in a geographical bias. Our time-scaled tree generated an estimated evolution rate of 3.05 × 10^−3^ for the near-complete genome, which is slightly lower than 3.8 × 10^−3^ in 2018 (Dyrdak et al., 2019), but higher than another estimate of 2.99 × 10^−3^ from 2017 (Eshaghi et al., 2017). Some patient sequences (patient 1, 4,12) appeared to have higher genetic diversity than average for the sampling date (Supplementary Figure S3; Supplementary Table S7), suggesting a possible higher level of viral evolution within these patients

The generation of high-quality near-complete genomes potentially allows the investigation of inter-and intra-host relations of SNV (Dyrdak et al., 2019; Cassidy et al., 2022). As seen in the SARS-CoV-2 pandemic, variants may explain changes in transmissibility and pathogenicity (Yang et al., 2022). We report a high level of variation within our study, particularly in the VP1 and 3C (transcribing the EV-D68-encoded protease) genes, however in this small cohort, any link between SNV and clinical outcome would not be statistically relevant. Moreover, coverage bias may have been introduced in our study with some samples generating a low volume of reads, while other samples obtaining sequencing depths higher than 100,000× in some regions. This coverage bias was particularly prominent in regions with overlapping primer fragments (Supplementary Figure S2). As a result, we applied a coverage cut-off of 100,000× to reduce the chances of sequencing or PCR errors which could be called wrongly due to the sheer number of reads. Additionally, although continuous improvements in ONT error rate have been made over the recent years (Wick et al., 2021), the overall high error rate compared to Illumina sequencing could also be a limitation. Variants with the highest frequency (in 90% of patient sequences) were subsequently revealed to be highly conserved within the B3 and A2 subclades (>90% in our reference database).

The high number of variants within the hypervariable VP1 gene could highlight the impact of immune pressure and its continual influence to shape this region (Opanda et al., 2014; Eshaghi et al., 2017). Other studies have identified rapidly evolving neutralizing epitopes in the VP1 gene, particularly in the BC and DE loops, with higher amino acid substitution rates than the rest of the genome (Hodcroft et al., 2022). With the emergence of new EV-D68 strains, given the high genetic diversity observed over time (Figures 2, 3), coupled with declining immunity (Kramer et al., 2018), it could indicate a growing susceptible population. At the same time, the requirement of re-amplification in some sample fragments could increase the chances for PCR driven mutations (Salk et al., 2018).

Finally, we show evidence of high levels of episodic positive diversifying selection events within the EV-D68 population and within our patient dataset scattered along the genome, particularly in the 3C and VP1 genes (referred to as “hotspot” regions; Muslin et al., 2019). As our patient samples are the most recently deposited (near-full) EV-D68 sequences, it could highlight the areas which are most likely being selected in the future. Interestingly, the 3C gene has a central role in inhibiting antiviral immunity (Xiang et al., 2015). It has been speculated that the now dominant B3 subclade has arose from diversifying selection and likely represents a newly emerging strain, as opposed to evolving from the B1 or B2 clades (Wang et al., 2017; Hodcroft et al., 2022). Given the strong selection pressure observed (in this case positive diversifying selection) of subclade B3, along with a potentially increasing naïve population, it could have driven its predominance since 2018.

## 5. Conclusion

We report an off-season EV-D68 upsurge likely caused by highly diverse subclades and associated with severe respiratory and neurological disease, particularly in children and immunocompromised adults. It could be that the positive episodic or diversifying selection, along with persistent yet undetected circulation may be driving EV-D68 evolution, however at this point this is still a working hypothesis. Furthermore, high levels of non-synonymous mutations, particularly in the surface proteins, could suggest monitoring closely for potential challenges with routine Sanger sequencing. Applying WGS revealed an dynamic picture of EV-D68, highlighting evolutionary patterns. With the re-emergence of EV-D68 following the COVID-19 lockdown and poliovirus recently detected in non-epidemic countries, it is important to closely monitor this virus which may become a threat to public health.

## Data availability statement

The original contributions presented in the study are publicly available. This data can be found here: https://www.ncbi.nlm.nih.gov/bioproject/PRJNA900246.

## Author contributions

HC: study design, laboratory and data analysis, writing-original draft preparation, and writing-reviewing and editing. EL-F: data analysis and writing-reviewing and editing. LS: study design, laboratory and data analysis, and writing-reviewing and editing. CL-B and HGMN: conceptualization, editing, and supervision. All authors contributed to the article and approved the submitted version.

## Funding

Hayley Cassidy and Leonard Schuele received funding from the European Union’s Horizon 2020 research and innovation program, under the Marie Sklodowska-Curie grant agreement 713660 (MSCA-COFUND-2015-DP “Pronkjewail”). Erley Lizarazo-Forero was funded by Quality Control Molecular Diagnostics (QCMD, Glasgow, Scotland) under an unrestricted grant.

## Conflict of interest

The authors declare that the research was conducted in the absence of any commercial or financial relationships that could be construed as a potential conflict of interest.

## Publisher’s note

All claims expressed in this article are solely those of the authors and do not necessarily represent those of their affiliated organizations, or those of the publisher, the editors and the reviewers. Any product that may be evaluated in this article, or claim that may be made by its manufacturer, is not guaranteed or endorsed by the publisher.

## Footnotes

^1^ dx.doi.org/10.17504/protocols.io.bdp8i5rw

^2^ https://www.rivm.nl/mpf/typingtool/enterovirus/
