# Supplementary material for: Off-season circulation and characterization of enterovirus D68 with respiratory and neurological presentation using whole-genome sequencing
Source: Front Microbiol. 2023 Feb 9;13:1088770. doi: 10.3389/fmicb.2022.1088770 (PMC9947850; doi:10.3389/fmicb.2022.1088770)
Supplement: Supplementary file 1 [file Data_Sheet_1.docx]

Supplementary Material

# Supplementary Materials and Methods

## Validation

Initially, a 1/10 serial dilution of the enterovirus D68 (EV-D68) culture was created using viral transport medium. The serial dilutions were isolated using the easyMAG (bioMérieux, Inc., Marcy l’Etoile, France) and eluted in 110µL. Lysis buffer served as a negative control. An EV-D68 specific real-time reverse transcriptase PCR (RT-qPCR) was performed to aid in selecting a representative Ct value (Ct 26) for the positive control. Near-full genome amplification was then performed as specified in the Materials and Methods section in the manuscript. Complementary DNA was run on the Agilent 2200 TapeStation system to confirm the sizes of the fragments (average 1,800bps). The Rapid Sequencing Kit (SQK-RAD004) (ONT) was selected for library generation and sequenced on a Flongle (FLO-MIN106 R9.4.1) flow cell (ONT). Figure S1 illustrates the genome coverage pattern achieved for the positive control (Ct 26).

## Phylogenetic tree (Reference sequences)

Virus Pathogen Resource (https://www.viprbrc.org/brc/home.spg?decorator=vipr) was used to download near-full length references for phylogenetic analysis using the following parameters: human origin, between >7,000 and <8,000 and collected between 1962 and 2019. A total of 892 EV-D68 reference sequences were generated, along with the Fermon strain (accession number AY426531) (n=893). A total of 21 sequences were subsequently removed following an initial alignment using MAFFT (v7.471), due to poor sequence quality (Table S8). This was similarly performed previously (Duchêne et al. 2020). An additional 29 references were removed after a second alignment in the Nextstrain pipeline. The Nextstrain pipeline automatically removed sequences during tree refinement (n=12) and uploading to auspice (n=7) (Table S8). A total of 824 EV-D68 references were used in the phylogenetic tree and subsequent selection pressure analysis.

## References

Duchêne S. et al. (2020) ‘Bayesian Evaluation of Temporal Signal in Measurably Evolving Populations,’ Molecular Biology and Evolution, msaa163.

# Supplementary Tables

**Table S1.** Trimming parameters

| **Mapping to reference parameters** | **Value** |
| --- | --- |
| Guppy (MinKNOW interface) v6.0.1 | Standard parameters and selection of discard middle and require two barcodes |

**Table S2.** Mapping to the EV-D68 reference using CLC Genomics Workbench v21.0.5

| **Mapping to reference parameters** | **Values** |
| --- | --- |
| Masking mode | No masking |
| Match score | 1 |
| Mismatch cost | 2 |
| Cost of insertions and deletions | Linear gap cost |
| Insertion cost | 3 |
| Deletion cost | 3 |
| Length fraction | 0.7 |
| Similarity fraction | 0.8 |
| Global alignment | No |
| Non-specific match handling | Map randomly |
| Output mode | Create stand-alone read mappings |
| Create report | Yes |
| Collect unmapped reads | No |

**Table S3.** EV-D68 Nextstrain analysis using the augur pipeline v7.0.2 and TimeTree v0.8.1

| augur align –sequences --output |
| --- |
| augur tree –alignment –method iqtree –output --substitution-model  GTR+G --nthreads 16 |
| augur refine –tree –alignment –metadata --output-tree --outputnode-  data --timetree --coalescent  skyline --covariance --precision 3 --date-confidence --date-inference  marginal --gen-per-year 50 --branch-length-inference auto --clockfilter-  iqd 4 --divergence-unit mutations --root AY426531_Fermon |
| augur export v2 –tree –metadata --node-data --  output |

**Table S4.** Selection analysis using HYPHY v2.5.32

| **Parameters** | **Value** |
| --- | --- |
| Selection Analysis | MEME |
| Genetic code | Universal code |
| Branches to select for analysis | Include all branches in the analysis |
| P-value threshold | Default (0.1) |
| Perform parametric bootstrap resampling | Default (50) |
| Nucleotide substitution biases | GTR |
| Reduce zero-length branches | Yes |
| Perform branch length re-optimization under the full codon model | Default ( yes) |

**Table S5.** Single nucleotide variation using the Fixed Ploidy Variant Detection tool on CLC Genomics Workbench v21.0.5

| **Fixed Ploidy Variant Detection** | **Values** |
| --- | --- |
| Ploidy | 1 |
| Required variant probability (%) | 90 |
| Ignore positions with coverage above | 100000 |
| Restrict calling to target regions |  |
| Ignore broken pairs | true |
| Ignore non-specific matches | Reads |
| Minimum read length | 20 |
| Minimum coverage | 100 |
| Minimum count | 90 |
| Minimum frequency (%) | 90 |
| Base quality filter | false |
| Neighborhood radius | 5 |
| Minimum central quality | 20 |
| Minimum neighborhood quality | 15 |
| Read direction filter | false |
| Direction frequency (%) | 5 |
| Relative read direction filter | false |
| Significance (%) | 1 |
| Read position filter | false |
| Significance (%) | 1 |
| Remove pyro-error variants | false |
| In homopolymer regions with minimum length | 3 |
| With frequency below | 0.8 |

**Table S6.** Average read length using CLC Genomics Workbench v21.0.5

| **Sample name** | **Average read length** |
| --- | --- |
| EVD68_GR_01_22.11.19 | 533.78 |
| EVD68_GR_02_23.11.19 | 2,012.71 |
| EVD68_GR_03_23.11.19 | 713.44 |
| EVD68_GR_04_28.11.19 | 1,986.83 |
| EVD68_GR_05_02.12.19 | 1,220.10 |
| EVD68_GR_06_08.12.19 | 1,964.76 |
| EVD68_GR_07_09.12.19 | 1,969.33 |
| EVD68_GR_08_11.12.19 | 1,970.91 |
| EVD68_GR_09_14.12.19 | 1,958.64 |
| EVD68_GR_10_14.12.19 | 611.36 |
| EVD68_GR_11_17.12.19 | 1,978.86 |
| EVD68_GR_12_25.12.19 | 1,885.75 |
| EVD68_GR_13_13.01.20 | 1,252.64 |
| EVD68_GR_14_20.01.20 | 1,460.45 |
| EVD68_GR_15_21.01.20 | 369.21 |
| EVD68_GR_16_29.01.20 | 525.33 |
| EVD68_GR_17_19.02.20 | 624.46 |
| EVD68_GR_18_12.03.20 | 1,655.99 |
| EVD68_GR_19_13.03.20 | 1,683.92 |
| EVD68_GR_20_19.03.20 | 856.96 |

**Table S7.** EV-D68 genetic divergence in off-season upsurge samples using TempEst v2.7.0

| **Sample name*^1^** | **Date** | **Distance** | **Residual** | **Regression line** |
| --- | --- | --- | --- | --- |
| EVD68_GR_01_22.11.19 | 2019.057555 | 0.211451353 | 0.001686948 | Above |
| EVD68_GR_02_23.11.19 | 2019.060295 | 0.210464853 | 6.91E-04 | Below |
| EVD68_GR_03_23.11.19 | 2019.060295 | 0.207754783 | -0.00201948 | Below |
| EVD68_GR_04_28.11.19 | 2019.073994 | 0.211845673 | 0.002022121 | Above |
| EVD68_GR_06_08.12.19 | 2019.019201 | 0.209611123 | -1.53E-05 | Below |
| EVD68_GR_07_09.12.19 | 2019.021941 | 0.209137813 | -4.98E-04 | Below |
| EVD68_GR_08_11.12.19 | 2019.02742 | 0.209309453 | -3.47E-04 | Below |
| EVD68_GR_09_14.12.19 | 2019.035639 | 0.209292013 | -3.94E-04 | Below |
| EVD68_GR_10_14.12.19 | 2019.035639 | 0.209000363 | -6.85E-04 | Below |
| EVD68_GR_11_17.12.19 | 2019.043858 | 0.209204093 | -5.11E-04 | Below |
| EVD68_GR_12_25.12.19 | 2019.065776 | 0.218156203 | 0.008362218 | Above |
| EVD68_GR_13_13.01.20 | 2020.032789 | 0.211711133 | -0.001562237 | Below |
| EVD68_GR_14_20.01.20 | 2020.051914 | 0.209684743 | -0.003657442 | Below |
| EVD68_GR_15_21.01.20 | 2020.054647 | 0.210319973 | -0.003032043 | Below |
| EVD68_GR_16_29.01.20 | 2020.076505 | 0.209043363 | -0.0043873 | Below |
| EVD68_GR_17_19.02.20 | 2020.049184 | 0.209190503 | -0.004141858 | Below |
| EVD68_GR_18_12.03.20 | 2020.03006 | 0.210008493 | -0.00325506 | Below |
| EVD68_GR_19_13.03.20 | 2020.032793 | 0.210333013 | -0.00294037 | Below |
| EVD68_GR_20_19.03.20 | 2020.049186 | 0.210241493 | -0.003090875 | Below |

*1 EV-D68 patients (n=19). The Nextstrain pipeline automatically removed problematic sequences during alignment, this included EVD68_GR_05_02.12.19. This alignment was used to generate the maximum likelihood tree without a molecular clock for TempEst v2.7.0.

**Table S8.** Excluded reference sequences prior to phylogenetic analysis

| **Removed after  initial sequence alignment (MAFFT) due to poor quality** | **Nextstrain pipeline (automatic removal)** | | | **Total** |
| --- | --- | --- | --- | --- |
|  | **Removed after  sequence alignment** | **Removed after  tree refine** | **Removed after  uploading to auspice** |  |
| MF045413 | MF045413 | KU844179 | MN240496 |  |
| MF045414 | MF045414 | KU844178 | MH341730 |  |
| MF045415 | MF045415 | MN240508 | MH341732 |  |
| MF045416 | MF045416 | MN240507 | MH341734 |  |
| MF045417 | MF045417 | MN240498 | MK419062 |  |
| MF045418 | MF045418 | KX433167 | MK419066 |  |
| MF045419 | MF045419 | KX261825 | MK419077 |  |
| MF045422 | MF045422 | KX255412 |  |  |
| KX351809 | KX351809 | KM892500 |  |  |
| KX351826 | KX351826 | KT725431 |  |  |
| KX433155 | KX433155 | KT803589 |  |  |
| KX433156 | KX433156 | MK419060 |  |  |
| KX433157 | KX433157 |  |  |  |
| KX433160 | KX433160 |  |  |  |
| KX433162 | KX433162 |  |  |  |
| KX433163 | KX433163 |  |  |  |
| KX789263 | KX789263 |  |  |  |
| MH674118 | MH674118 |  |  |  |
| MH674126 | MH674126 |  |  |  |
| MN245445 | MH674132 |  |  |  |
| KU242684 | MN245445 |  |  |  |
|  | MN935869 |  |  |  |
|  | KU242684 |  |  |  |
|  | MN240501 |  |  |  |
|  | MN240499 |  |  |  |
|  | MN240510 |  |  |  |
|  | KT285484 |  |  |  |
|  | KY767820 |  |  |  |
|  | MK419061 |  |  |  |
| **21** | **29** | **12** | **7** | **69** |

**Table S9.** Distribution of single nucleotide variants per nucleotide position per each off-season patient sample (n=20) compared to the Fermon strain (GenBank accession number AY426531). Mutation including the gene, type and nucleotide(s) change are depicted in the excel document. Relevant clinical details, including clinical presentation, sample material, Ct value and subclade type are also shown. **Abbreviations**: nt; nucleotide, MNV; multiple nucleotide variation, SNV; single nucleotide variation, AFM; acute flaccid myelitis, CSF; cerebrospinal fluid, Ct; cycle threshold

**Table S10.** Selection pressure on individual codon sites following a Mixed Effects Model of Evolution applied on Datamonkey (v1.6.0) (PP value <0.05).

| **Condon site (Patients only)*^1^** | **Condon site (Patients and references)*^2^** | | | |
| --- | --- | --- | --- | --- |
| 232 | 19 | 591 | 846 | 1708 |
| 353 | 58 | 593 | 956 | 1721 |
| 771 | 109 | 595 | 1044 | 1790 |
| 948 | 143 | 607 | 1055 | 1797 |
| 1433 | 194 | 613 | 1125 | 1852 |
| 1606 | 220 | 649 | 1126 | 1856 |
| 1625 | 283 | 669 | 1209 | 1869 |
| 1657 | 377 | 700 | 1222 | 2068 |
| 1899 | 405 | 709 | 1314 | 2187 |
| 1965 | 419 | 746 | 1535 |  |
|  | 523 | 771 | 1537 |  |
|  | 524 | 788 | 1542 |  |
|  | 551 | 818 | 1569 |  |
|  | 553 | 819 | 1622 |  |
|  | 584 | 841 | 1623 |  |

*1 EV-D68 patients (n=19). The Nextstrain pipeline automatically removed problematic sequences during alignment, this included EVD68_GR_05_02.12.19. This alignment was used for selection analysis on HYPHY v2.5.32. *2 Reference genomes (n=824), along with EV-D68 patients (n=19)

# Supplementary Figures


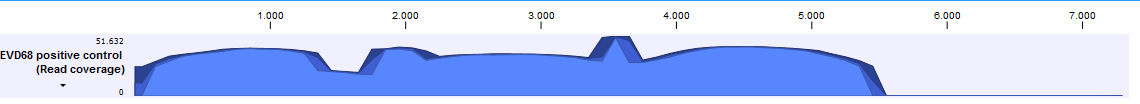

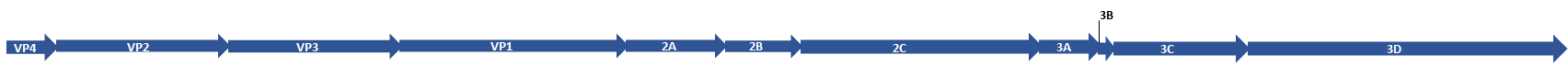


**Supplementary Figure 1.** Genome coverage pattern achieved in the positive control (Ct 26)


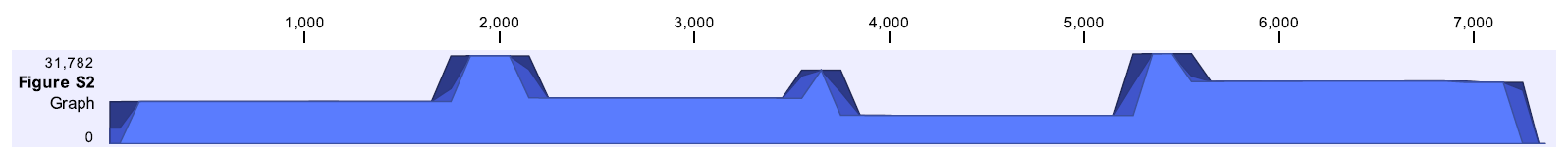

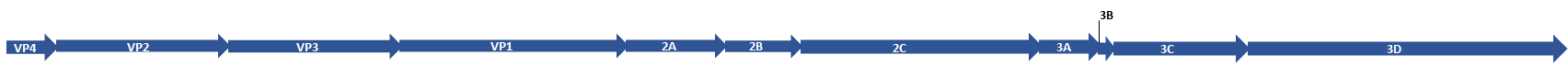


**Supplementary Figure 2.** Example of genome coverage pattern in one of the clinical samples EVD68_GR_09_14.12.19 (Ct 19)


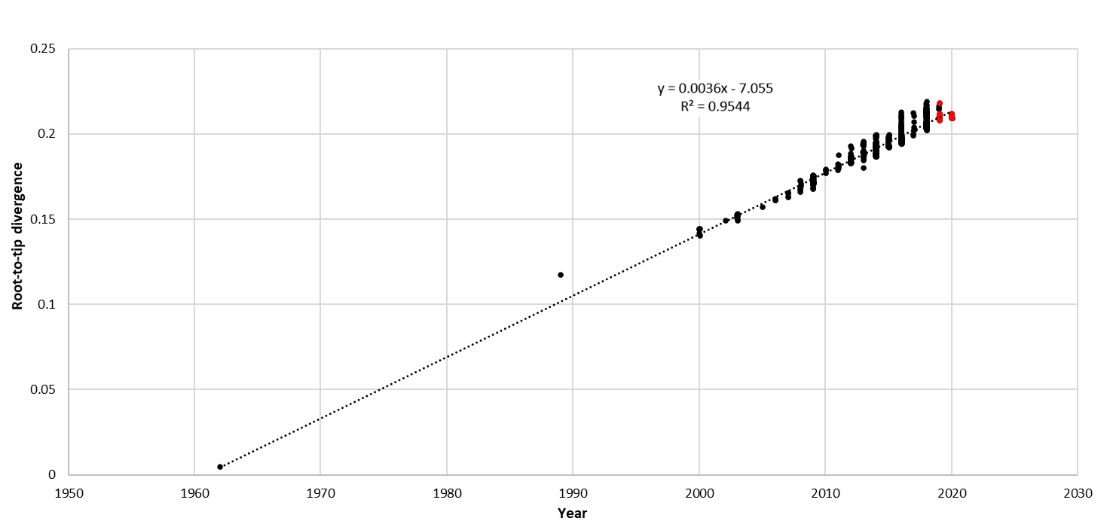


**Supplementary Figure 3.** EV-D68 genetic divergence. A root-to-tip regression analysis was generated using TempEST. The x-axis corresponds to the year of sampling and the y-axis corresponds to branch distances of the phylogenetic tree without a molecular clock (in units of substitution per site). Patient sequences are represented by red dots (n=19). The references are represented by black dots (n=824). Sequences above the regression line indicate higher divergence for the sampling date, whereas sequences below the regression line indicate less divergence for the sampling date. Table S7 provides an overview of patient root-to-tip regressions. While patient numbers 1, 4 and 12 appear to be above the regression line, indicating higher divergence for the sampling date, the remaining patient sequences are below the regression line, indicating lower divergence for the sampling date (Table S7)


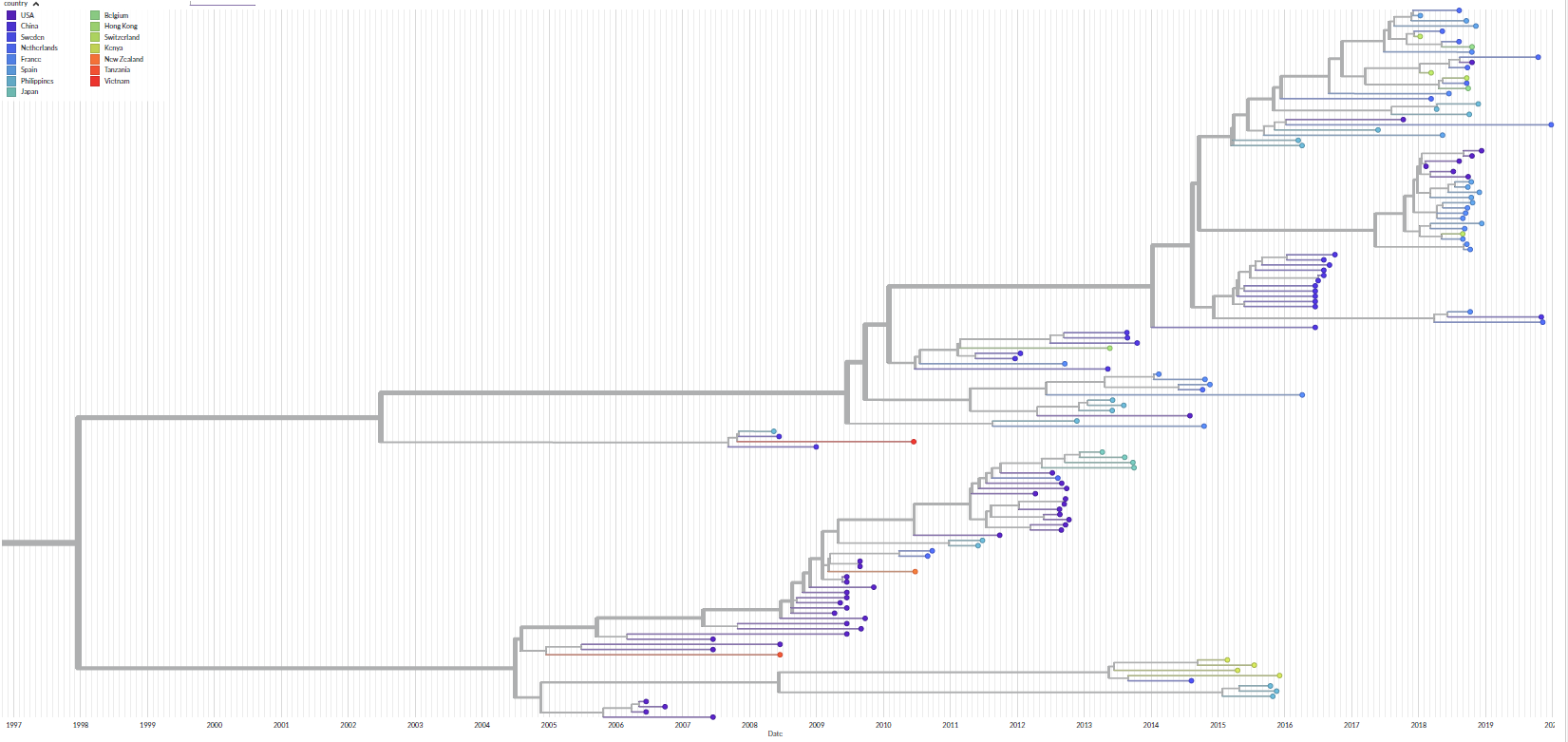

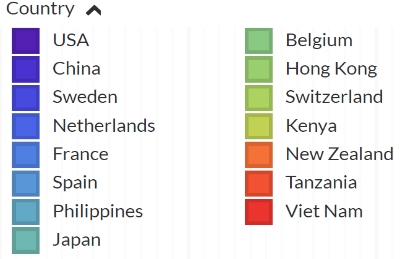


A2

A1

n=1

**Supplementary Figure 4.** Time-scaled phylogenetic tree of EV-D68 clade A1 and A2 near-full genomes (zoomed in) by year. The red arrow highlights our EV-D68 clade A2 patient. The augur pipeline implemented in Nextstrain was run using the aligner MAFFT and the phylodynamic package TreeTime, followed by visualization using auspice. A coalescent skyline model with a strict molecular clock and rooted using the Fermon strain (AY426531). Enterovirus sequences are highlighted according to country.

n=5

n=12


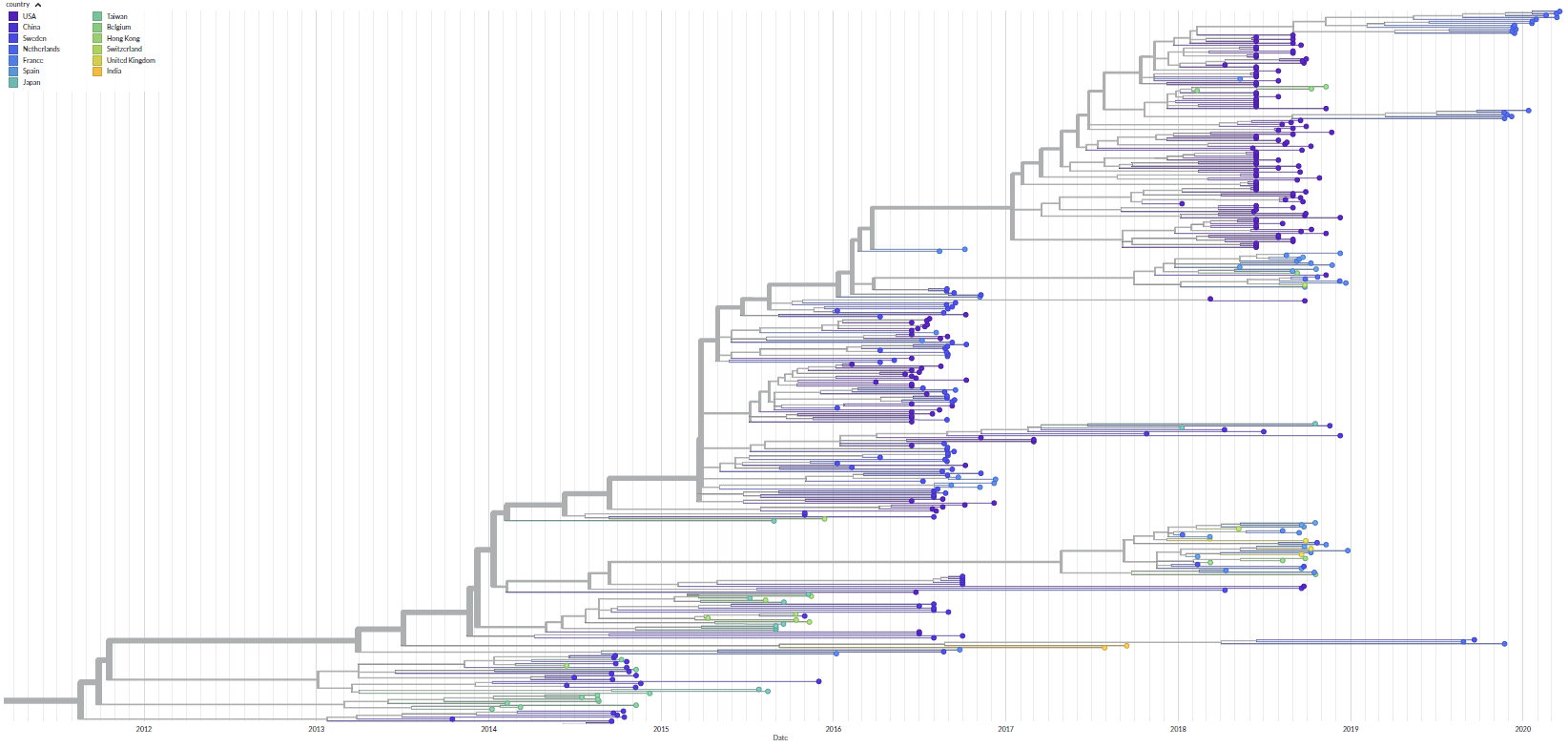


n=1


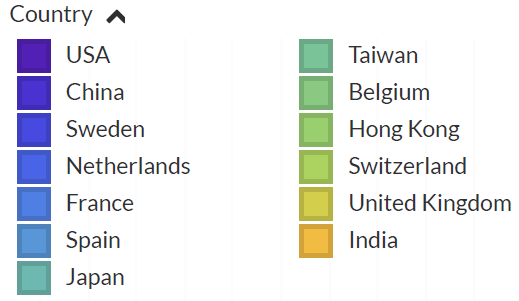


**Supplementary Figure 5.** Time-scaled phylogenetic tree of EV-D68 clade B3 near-full genomes (zoomed in). The red arrows highlight our EV-D68 clade B3 patients. The augur pipeline implemented in Nextstrain was run using the aligner MAFFT and the phylodynamic package TreeTime, followed by visualization using auspice. A coalescent skyline model with a strict molecular clock and rooted using the Fermon strain (AY426531). Enterovirus sequences are highlighted according to country.


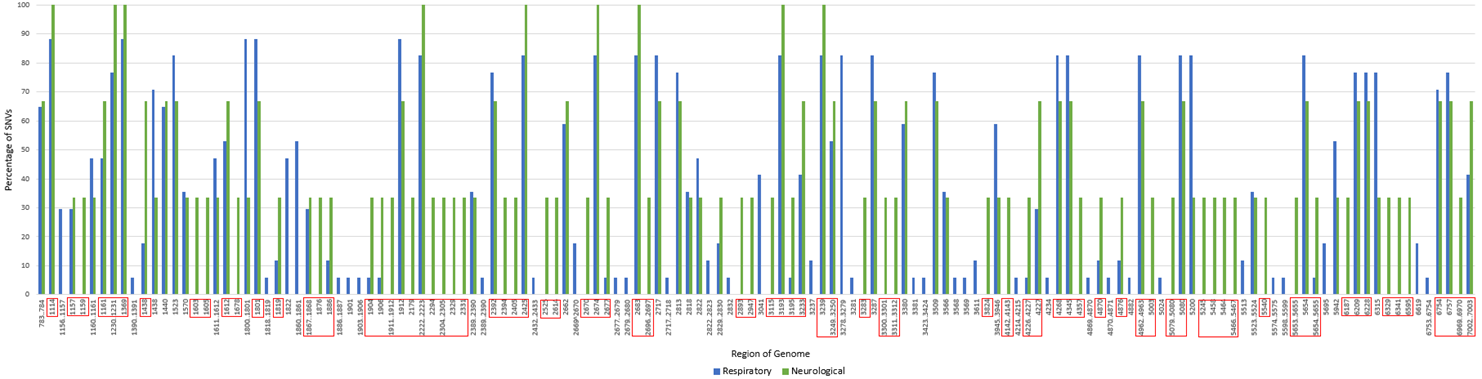


**Supplementary Figure 6.** Bar graph depicting the percentage of SNV within the patients with respiratory (n=17) (in blue) and neurological (n=3) presentation (in green) with the corresponding region in the genome. Red boxes represent the regions which contain SNV from Clade A2. For example, 65% of patients with a respiratory presentation had a SNV at genome region 783-784.
